# Wogonin Induces Reactive Oxygen Species Production and Cell Apoptosis in Human Glioma Cancer Cells

**DOI:** 10.3390/ijms13089877

**Published:** 2012-08-08

**Authors:** Cheng-Fang Tsai, Wei-Lan Yeh, Ssu Ming Huang, Tzu-Wei Tan, Dah-Yuu Lu

**Affiliations:** 1 Department of Biotechnology, Asia University, Wufeng, Taichung County 41354, Taiwan; E-Mail: tsaicf@asia.edu.tw; 2 Cancer Research Center, Department of Medical Research, Changhua Christian Hospital, Changhua 500, Taiwan; E-Mail: ibizayeh0816@hotmail.com; 3 Department of Colorectal Surgery, Buddhist Tzu Chi General Hospital, Taichung Branch, Taichung 427, Taiwan; E-Mail: szuming@hotmail.com; 4 Graduate Institute of Biotechnology, National Chung Hsing University, Taichung 402, Taiwan; 5 Department of Pharmacology, China Medical University, Taichung 40402, Taiwan; E-Mail: twtan@mail.cmu.edu.tw; 6 Graduate Institute of Neural and Cognitive Sciences, China Medical University, Taichung 40402, Taiwan

**Keywords:** ROS, apoptosis, wogonin, glioma, ER stress

## Abstract

Glioma is the most common primary adult brain tumor with poor prognosis because of the ease of spreading tumor cells to other regions of the brain. Cell apoptosis is frequently targeted for developing anti-cancer drugs. In the present study, we have assessed wogonin, a flavonoid compound isolated from *Scutellaria baicalensis* Georgi, induced ROS generation, endoplasmic reticulum (ER) stress and cell apoptosis. Wogonin induced cell death in two different human glioma cells, such as U251 and U87 cells but not in human primary astrocytes (IC 50 > 100 μM). Wogonin-induced apoptotic cell death in glioma cells was measured by propidine iodine (PI) analysis, Tunnel assay and Annexin V staining methods. Furthermore, wogonin also induced caspase-9 and caspase-3 activation as well as up-regulation of cleaved PARP expression. Moreover, treatment of wogonin also increased a number of signature ER stress markers glucose-regulated protein (GRP)-78, GRP-94, Calpain I, and phosphorylation of eukaryotic initiation factor-2α (eIF2α). Treatment of human glioma cells with wogonin was found to induce reactive oxygen species (ROS) generation. Wogonin induced ER stress-related protein expression and cell apoptosis was reduced by the ROS inhibitors apocynin and NAC (*N*-acetylcysteine). The present study provides evidence to support the fact that wogonin induces human glioma cell apoptosis mediated ROS generation, ER stress activation and cell apoptosis.

## 1. Introduction

Glioblastomas are one of the most lethal types of primary central nervous system tumors, and their biological features make successful treatment very difficult. Moreover, glioblastomas generally prove refractory to treatment by surgery, irradiation, and conventional chemotherapy. Their abnormal biological features lead to uncontrolled growth, invasiveness, and angiogenesis, and ultimately facilitate cell proliferation and affect survival [1–5]. These dysregulated pathways provide the basis for designing molecular-targeted therapy for treatment of gliomas.

Endoplasmic reticulum (ER) is an organelle in the secretory pathways, and plays a central role in lipid synthesis, protein folding and modification. However, protein folding in the ER is impaired under a variety of toxic insults, including hypoxia, failure of protein synthesis, protein misfolding, and Ca^2+^ overload, and can result in ER stress-related events [6,7]. There is increasing evidence that ER stress plays a crucial role in the regulation of apoptosis. It has been reported that ER stress triggers several specific signaling pathways, such as ER-associated protein degradation and the unfolded protein response (UPR) [8,9]. The UPR induces the expression of ER-resident chaperones, such as GRP(glucose-regulated protein)-78 and GRP-94 [10]. GRPs are the most abundant glycoproteins in the ER and play critical roles in ER regulation. The protective functions of GRP protein have also been observed in resistance to radiation in cancer cells [11]. On the other hand, several pro-apoptotic factors like CHOP/GADD153, and pro-apoptotic Bcl-2 family members like Bax, have been also shown to be involved in ER stress-induced cell death. CHOP/GADD153 is apparently a pro-apoptotic transcription factor induced during ER stress [12–15]. The eukaryotic translation initiation factor 2 alpha (eIF2α) phosphorylation is a highly conserved point of the molecule that adapts cells to ER stress [9,16]. It provides stress resistance by arresting protein translation and induction of stress-inducible cytoprotective proteins. Furthermore, reactive oxygen species (ROS) generation appears to be triggered by the activation of the mitochondrial-dependent cell death pathway through the pro-apoptotic Bcl-2 proteins Bax or Bak, and is further transformed sequentially into more toxic ROS, such as hydrogen peroxide reactive oxygen species, which consequently induce cell death [17,18].

Wogonin, 5,7-dihydroxy-8-methoxyflavone, is one of the major flavonoids found in the root of the baicalensis Georgi, which is widely used in treating allergic and inflammatory diseases [19]. It has been reported that wogonin suppresses lipopolysaccharide (LPS)-induced inflammatory cytokine release in macrophages and microglial cells [20–22], and reduces cell migration activity on microglial cells [23]. Moreover, treatment with wogonin alleviates inflammatory responses caused by skin inflammation and carrageenan-induced hindpaw edema in animal studies [24,25]. Wogonin also exerts neuroprotective effects by inhibition of neuronal cell death and reducing microglia activation in global ischemia and excitotoxic injury models [21]. Furthermore, wogonin also reduced ischemic neuronal injury in a mice model [26,27] and in cell culture [28,29]. Recent report also revealed that wogonin improves histological and functional outcomes in experimental traumatic brain injury [30]. Flavonoids are naturally occurring polyphenolic compounds present in fruits, vegetables and some medicinal plants, and are recognized to be cancer preventive agents or anti-neoplastic agents. Wogonin, one of the active flavones of the most popular Chinese herb Huang-Qin (*Scutellaria baicalensis* Georgi), induces apoptosis in a wide spectrum of human tumor cells [31], such as osteosarcoma [32], leukemia [33], breast cancer [34] as well as glioma [35]. Importantly, *Scutellaria* extracts have been successfully tested in patients with advanced breast cancer in early clinical trials [36,37]. In addition, at doses lethal to tumor cells, wogonin showed no or little toxicity for normal cells and had also no obvious toxicity in animals [34,38–41]. Despite evidence indicating the benefits of wogonin treatment for neurological diseases, there is a lack of data describing the anti-tumor activity of wogonin on the central nervous system. Our study indicates that wogonin induces human glioma cell apoptosis mediated ROS generation, ER stress activation and cell apoptosis.

## 2. Materials and Methods

### 2.1. Materials

Wogonin, NAC, apocynin and eIF2 inhibitor were obtained from Sigma-Aldrich (St. Louis, MO, USA). Fetal bovine serum (FBS), Dulbecco’s modified Eagle’s medium (DMEM), and OPTI-MEM were purchased from Gibco BRL (Invitrogen Life Technologies, Carlsbad, CA, USA). Primary antibodies against cleaved caspase 3, and phosphorylation of eIF2α were purchased from Cell Signaling and Neuroscience (Danvers, MA, USA). Primary antibodies specific for calpain 1, GRP78, GRP94, PARP-1/2, pro-caspase 3, pro-caspase 9, and β-actin were purchased from Santa Cruz Biotechnology (Santa Cruz, CA, USA). Propidium iodide (PI), and 2′,7′-dichlorodihydrofluorescein diacetate (H_2_DCFDA) were obtained from Molecular Probes (Eugene, OR, USA).

### 2.2. Cell Culture

U87 and U251 cells originated from a human brain glioma. All cell lines were purchased from the American Type Culture Collection (Manassas, VA, USA), and maintained in 75 cm^2^ flasks with DMEM.

Human primary astrocytes were purchased from Sciencell Research Laboratories (isolated from human cerebral cortex, Cat# 1800, Carlsbad, CA, USA) and were cultured in human astrocyte medium (Sciencell, Cat# 1801) on poly-l-lysine coated tissue culture dishes. Media was changed every three days and cells were passaged once a week at a 1:5 ratio.

All cells were cultured in medium supplemented with 10% FBS, 100 U/mL penicillin, and 100 mg/mL streptomycin at 37 °C, incubated in a humidified atmosphere consisting of 5% CO_2_ and 95% air.

### 2.3. MTT Assay

Cell viability was determined by 3-(4,5-dimethylthiazol-2-yl)-2,5-diphenyltetrazolium bromide (MTT) assay. After treatment with wogonin for 24 or 48 h, mediums were removed and washed with PBS. MTT (0.5 mg/mL) was then added to each well and the mixture was incubated for 2 h at 37 °C. MTT reagent was then replaced with DMSO (100 μL per well) to dissolve formazan crystals. After the mixture was shaken at room temperature for 10 min, absorbance was determined at 550 nm using a microplate reader (Bio-Tek, Winooski, VT, USA).

### 2.4. Quantification of Apoptosis by Flow Cytometry

Cells were treated with various concentrations of wogonin for 24 h and then washed with PBS. For sub-G1 determination, cells were fixed with 70% ethanol at room temperature and then re-suspended in PBS containing 50 μg/mL propidium iodide (PI), 100 μg/mL RNase A and 0.1% Triton X-100 for 30 min. Cells were immediately analyzed using FACScan and the Cellquest program (Becton Dickinson, Lincoln Park, NJ, USA).

Apoptosis was assessed by binding of annexin V protein to exposed phosphoserine (PS) residues at the surface of cells undergoing apoptosis. Cells were treated with wogonin for 24 h and then washed twice with PBS and re-suspended in staining buffer containing propidium iodide (PI, 10 μg/mL) and annexin V-FITC (2.5 μg/mL). Double-labeling was performed at room temperature for 10 min in darkness before flow cytometric analysis. Cells were immediately analyzed using FACScan and the Cellquest program (Becton Dickinson, Lincoln Park, NJ, USA).

Quantitative assessment of apoptotic cells was also conducted by the terminal deoxynucleotidyl transferase-mediated deoxyuridine triphosphate nick end labeling (TUNEL) method, which examines DNA-strand breaks during apoptosis with the BD ApoAlert™ DNA Fragmentation Assay Kit (Lincoln Park, NJ, USA). Cells were incubated with wogonin for the indicated time periods, trypsinized, fixed with 4% paraformaldehyde, and permeabilized with 0.1% Triton-X-100 in 0.1% sodium citrate. After undergoing washing, the cells were incubated with the reaction mixture for 60 min at 37 °C. The stained cells were then analyzed by flow cytometry.

### 2.5. Western Blot Analysis

The protocol of whole cell protein lysis was followed according to our previous report [42]. Briefly, cells were treated with wogonin for various time periods and then lysed with radioimmunoprecipitation assay buffer. Protein samples were separated by sodium dodecyl sulphate-polyacrylamide gels and transferred to polyvinyldifluoride membranes. The blots were probed with primary antibody for 1 h at room temperature and subsequently incubated with a secondary antibody for 1 h at room temperature. The blots were visualized by enhanced chemi-luminescence using Kodak X-OMAT LS film (Eastman Kodak, Rochester, NY, USA). The blots were subsequently stripped through incubation in stripping buffer and re-probed for β-actin as a loading control. Quantitative data were obtained using a computing densitometer and ImageQuant software (Molecular Dynamics, Sunnyvale, CA).

### 2.6. Reactive Oxygen Species (ROS) Assay

The production of ROS was assessed spectrofluorimetrically by oxidation of specific probes with 2′,7′-dichlorodihydrofluorescein diacetate (H_2_DCFDA). Cells were plated at six well-plates and exposed to wogonin for another 2 h. The cells were incubated with DHE (10 μM) or H_2_DCFDA (10 μM) for 30 min at 37 °C. The fluorescence intensity was measured with an excitation filter of 488 and 525 nm emission wavelengths using flow cytometry.

### 2.7. Statistics

The values given are means ± S.E.M. The significance of difference between the experimental group and control group was assessed by the Student’s *t* test. The difference was significant if the *p* value was <0.05.

## 3. Results and Discussion

### 3.1. Wogonin Induces Cell Apoptosis in Human Glioma Cells

**Figure 1:**
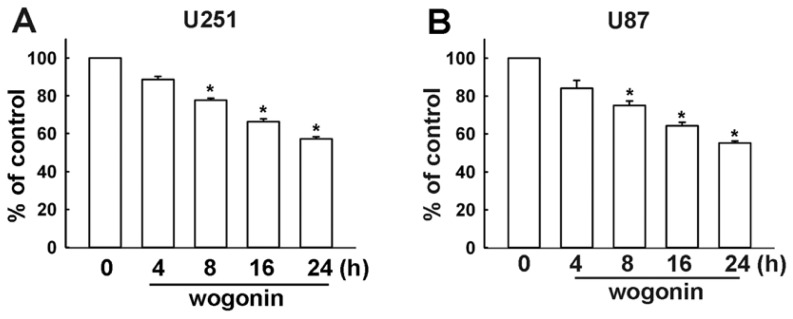
Wogonin induces cell death in human glioma cells. Wogonin-induced cell death of two different human glioma cells are shown in U251 (**A**) and U87 (**B**). Cells were incubated with wogonin (25 μM) for the indicated time periods, and cell viability was examined by MTT assay. Results are expressed as the means ± S.E.M. of at least three independent experiments. * *p* < 0.05 compared with the vehicle treatment group.

**Figure 2:**
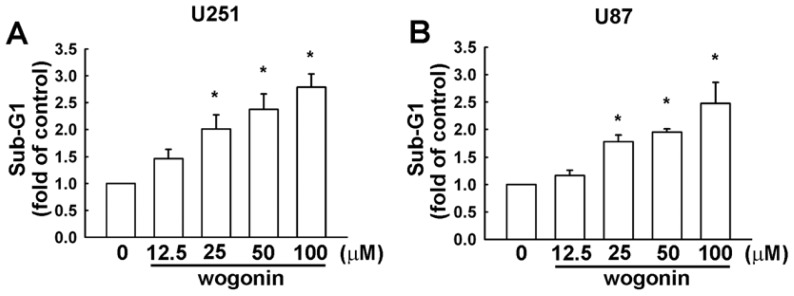
Wogonin induces cell apoptosis in human glioma cells. Cells were treated with various concentrations (12.5, 25, 50 or 100 μM) of wogonin for 24 h. The percentage of apoptotic cells (U251 (**A**) or U87 (**B**)) were analyzed by flow cytometry of propidine iodine (PI) staining. Results are expressed as the means ± S.E.M. of three independent experiments.

**Figure 3:**
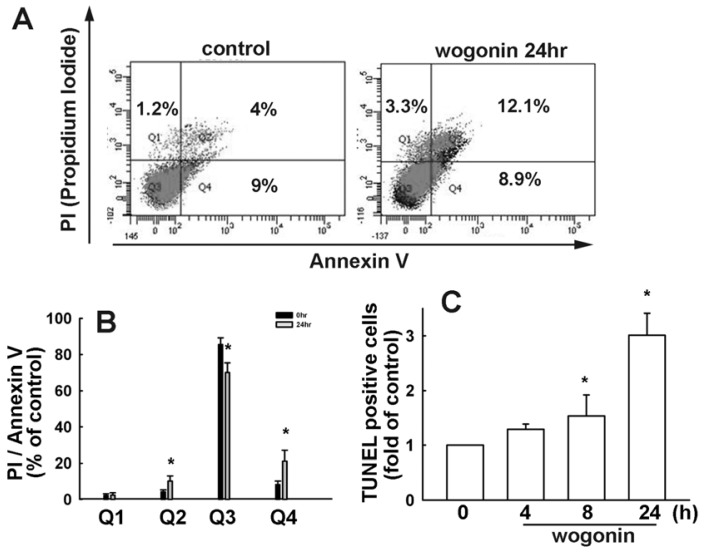
Wogonin induces cell apoptosis in U251 human glioma cells. (**A** and **B**) Cells were treated with wogonin (25 μM) for 24 h. The percentage of apoptotic cells was analyzed by flow cytometry of annexin V/PI double staining; (**C**) Cells were treated with wogonin (25 μM) for the indicated time periods, the TUNEL positive cells were examined by flow cytometry. Results are expressed as the means ± S.E.M. of at least three independent experiments. * *p* < 0.05 compared with the vehicle treatment group.

To investigate the cytotoxicity of wogonin on human glioma cells, we examined the effects on cell viability using MTT assay for 24 h. As shown in Figure 1, wogonin induced cell death in human U251 and U87 glioma cells time dependently, but not in primary human astrocytes (IC 50 > 100 μM; data not shown). Wogonin-induced cell death was also examined by evaluating the sub-G1 group using PI assay which was analyzed by flow cytometry. Wogonin induced sub-G1 arrest in U251 (Figure 2A) and U87 (Figure 2B) human glioma cells in a concentration-dependent manner. In the control condition, only 6.45 ± 0.39% and 5.33 ± 1.04% of U251 and C6 cells were positive cells, respectively. Next, we investigated whether wogonin induces cell death through an apoptotic mechanism. PI-annexin V double-labeling was used for the detection of PS (phosphoserine) externalization, a hallmark of early phase of apoptosis. As shown in Figure 3A,B, wogonin induced cell apoptosis in U251 human glioma cancer cells. We then investigated the effects of wogonin-induced apoptosis by using the TUNEL assay. Treatment with wogonin showed significant Tunnel positive activity in 8 and 24 h (Figure 3C). These data indicate that wogonin induces cell apoptosis on human glioma cancer cells.

### 3.2. Wogonin Induces Cell Apoptosis through Reactive Oxygen Species Production in U251 Human Glioma Cells

**Figure 4:**
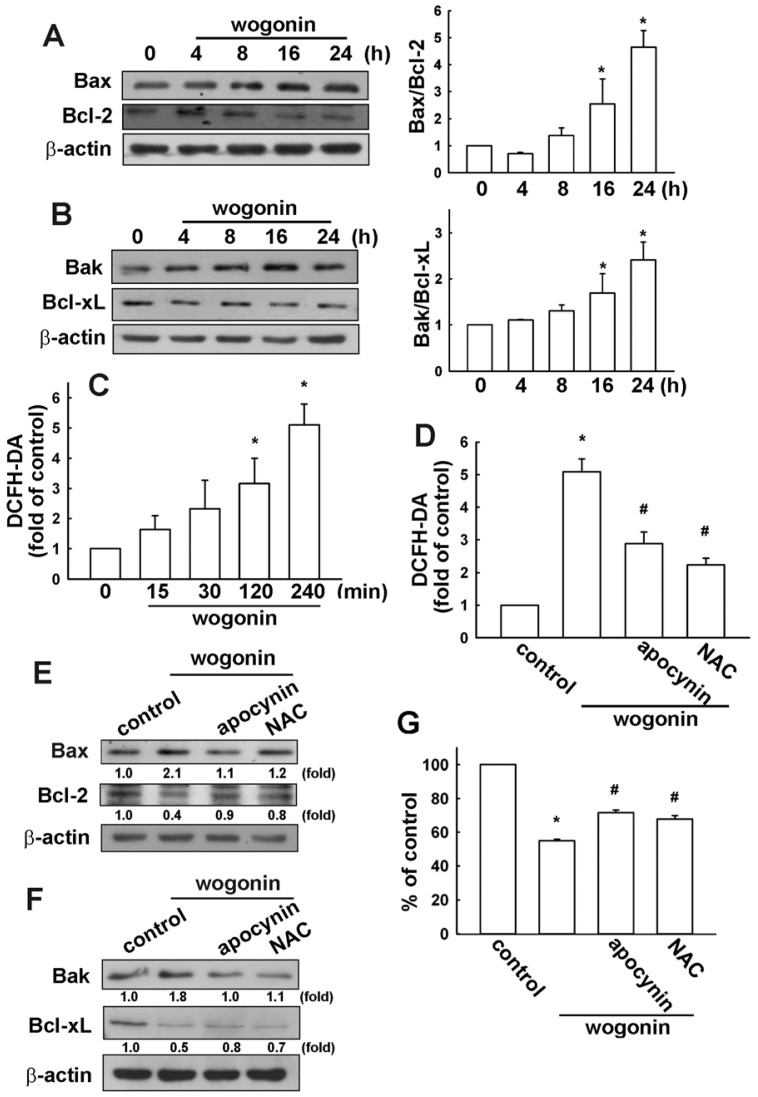
Wogonin increases reactive oxygen species generation in U251 human glioma cells. (**A** and **B**) Cells were incubated with wogonin (25 μM) for the indicated time periods, the Bax, Bcl-2, Bak and Bcl-xL expression were examined by Western blot analysis. Results are representative of three independent experiments. The ratios of the quantitative data are shown in the right panels; (**C**) Cells were incubated with wogonin (25 μM) for the indicated time periods (15, 30, 60 or 120 min); (**D**) Cells were pretreated with apocynin (10 μM) or *N*-acetylcysteine (NAC) (10 mM) for 30 min followed by stimulation with wogonin for 120 min. Reactive oxygen species (ROS) generation was determined using the fluorescence probes H_2_DCFH-DA. The production of ROS was examined by flow cytometry. Results are expressed as the mean ± S.E.M. of three independent experiments. * *p* < 0.05 compared with the control group. Cells were pretreated with apocynin (10 μM) or NAC (10 mM) for 30 min followed by stimulation with wogonin for 24 h, the protein expressions were examined by Western blot analysis (**E** and **F**), and cell viability was determined by MTT assay (G). Results are expressed as the mean ± S.E.M. of three independent experiments. * *p* < 0.05 compared with the control group. # *p* < 0.05 compared with the wogonin treatment group.

Bcl-2 family proteins play an important role in cancer cells apoptosis [43,44]. The Bcl-2 family can regulate mitochondrial membrane permeabilization. Bax protein mediates mitochondrial membrane permeabilization. Next, to determine whether wogonin induces cell apoptosis by triggering the mitochondrial apoptotic pathway, we measured the change in the expression of Bcl-2 family proteins. Treatment of U251 cells with wogonin induced Bax (Figure 4A) and Bak (Figure 4B) protein up-regulation. In addition, wogonin mild decreased the expression of Bcl-2 and Bcl-xL as well, which led to an increase in the pro-apoptotic/anti-apoptotic Bcl-2 family protein ratio. It has been reported that oxidative stress is implicated in the pro-apoptotic activities of cancer therapy [45,46]. Mitochondrial apoptotic pathway has been described as an important downstream signal of ROS in apoptotic cell death [47,48]. High levels of ROS can also induce apoptosis by triggering mitochondrial permeability transition pore opening, release of pro-apoptotic factors and activation of caspase-9 and caspase-3 [47,48]. It has been reported that inhibition of ROS by a ROS scavenger prevents camptothecin-induced apoptosis [49]. Here, we examined whether the ROS accumulation is involved in wogonin-induced glioma cell apoptosis. Wogonin induced an increase in intracellular ROS levels, as shown by H_2_DCF-DA staining which were analyzed by FACS detection assay (Figure 4C). Treatment with ROS inhibitors apocynin (a NADPH oxidase inhibitor) or NAC (a ROS scavenger) reduced wogonin-induced ROS production (Figure 4D) and, Bax and Bak expression (Figure 4E,F). Moreover, treatment with apocynin and NAC also dramatically reversed wogonin-induced cell death (Figure 4G). Additionally, apocynin and NAC did not affect cell viability at these concentrations (data not shown).

### 3.3. Wogonin Induces ER Stress-Related Protein Expression in U251 Human Glioma Cells

**Figure 5:**
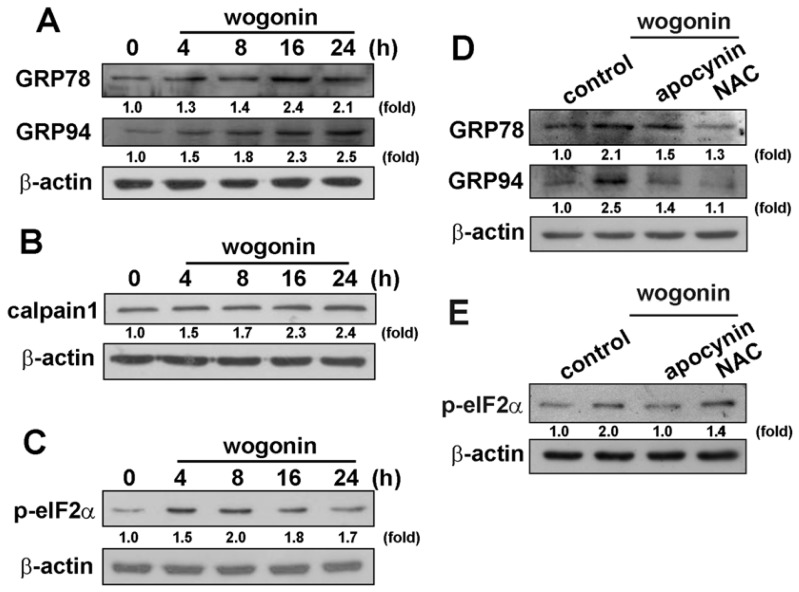
Wogonin increases endoplasmic reticulum (ER) stress-related protein activation in U251 human glioma cells. Cells were incubated with wogonin (25 μM) for the indicated time periods. GRP78 and GRP94 (**A**), calpain 1 (**B**) expression and eIF2α phosphorylation (**C**) were examined by Western blot analysis. Cells were pretreated with apocynin (10 μM) or NAC (10 mM) for 30 min followed by stimulation with wogonin for 24 h, GRP78 and GRP94 (**D**) expression, and eIF2α phosphorylation (**E**) were examined by Western blot analysis. Results are representative of three independent experiments.

ER stress is generally characterized by up-regulation of GRP78, GRP94, calpain 1, and phosphorylation of eukaryotic initiation factor-2α (eIF2α) [42]. Wogonin exposure caused a significant increase in the expression of GRP 78 and GRP 94 protein levels in a time-dependent manner (Figure 5A). We next determined whether the activity of calpain (calcium-dependent thiol proteases) would be induced by wogonin in human glioma cells time-dependently. As shown in Figure 5B, wogonin increased calpain 1 expression in U251 human glioma cells as well. Moreover, wogonin also induced the phosphorylation of eIF2α at Ser51 (Figure 5C). Treatment with ROS inhibitors apocynin or NAC reduced wogonin-induced GRP78 and GRP94 expression (Figure 5D) and phosphorylation of eIF2α (Figure 5E) as well. These results indicate that wogonin induces ER stress in human glioma cells.

### 3.4. Involvement of Caspase-9, Caspase-3 and PARP Cleavage in Wogonin Treatment in U251 Human Glioma Cells

**Figure 6:**
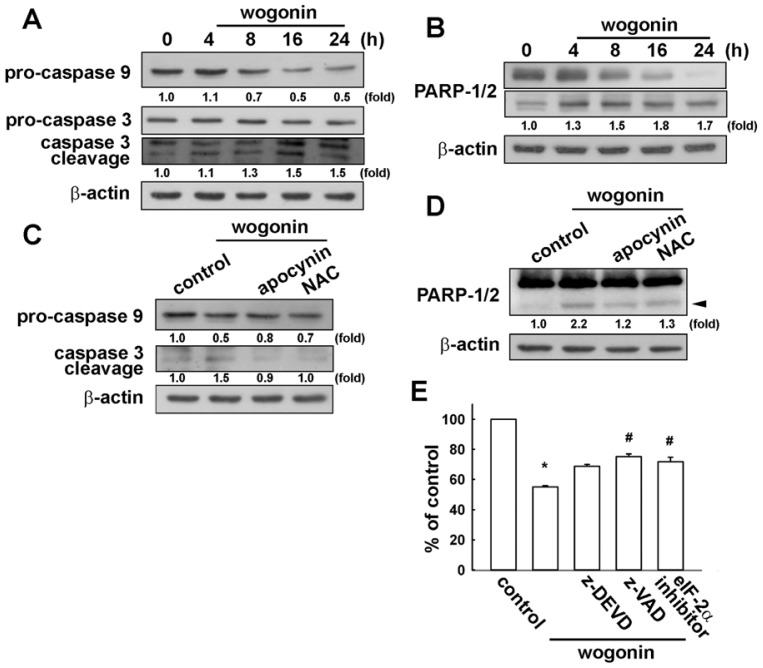
Wogonin induces activation of caspase-9, caspase-3 and PARP in U251 human glioma cells. Cells were incubated with wogonin (25 μM) for different time periods, levels of pro-caspase-9, pro-caspase-3 and cleaved caspase-2 (**A**) were examined by Western blot analysis. The expression of PARP cleaved form was also examined by Western blot analysis (**B**); (**C** and **D**) Cells were pre-incubated with apocynin and NAC for 30 min followed by treatment with wogonin for 24 h, the protein expressions were determined by Western blot analysis. Results are representative of at least three independent experiments; (**E**) Cells were pretreated with z-DEVD, z-VAD and eIF2 inhibitor for 30 min followed by stimulation with wogonin for 24 h, cell viability was determined by MTT assay.

One of the hallmarks of the apoptotic process is caspase activation, which represents both initiators and executors of death signals. It has been reported that ER stress–triggered apoptosis involves the activation of the intrinsic pathway of apoptosis involving the activity of caspase-9 and caspase-3 [50,51]. Treatment of wogonin also increased procaspase-3 degradation and caspase-3 cleaved form expression in U251 cells (Figure 6A). Upstream procaspase-9 is also degraded upon wogonin treatment in U251 cells (Figure 6A). Notably, wogonin also increased cleaved-PARP expression time-dependently (Figure 6B). Treatment with ROS inhibitors reduced wogonin-induced pro-caspase-9 degradation and caspase-3 cleavage (Figure 6C), and cleaved-PARP expression (Figure 6D) as well. Furthermore, treatment with pan-caspase inhibitor z-DEVD, caspase-3 inhibitor z-VAD and eIF2 inhibitor alleviated wogonin-induced cell death. These results suggest that wogonin triggers ROS generation, ER stress and induces cell apoptosis in human glioma cells.

## 4. Discussion and Conclusions

ROS plays an important role in the regulation of cellular functions such as cell proliferation, differentiation and immune responses [52]. ROS are produced through a variety of cellular events and exogenous sources, however, excessive production of ROS causes oxidative stress, which contributes to adverse events including tissue dysfunction and neuronal cell death [53,54]. Increasing evidence demonstrates that the accumulation of ROS is correlated with the apoptotic response induced by several chemotherapy agents. Importantly, chemotherapeutic drugs are selectively toxic to cancer cells since cancer cells appear to produce ROS at a greater rate than normal cells [55]. Accumulating results show that ROS is an important mediator in wogonin-induced apoptosis in human cancer cells, such as breast cancer cells [56], malignant T cells [39,41], and osteosarcoma [32], but not in normal cells even at the higher tested concentration (100 μM) [32,39,41,57,58]. Therefore, previous reports reveal that wogonin can be a potential antitumor drug without cytotoxicity in normal cells. Our study showed that wogonin can elevate the levels of intracellular ROS in human glioma cells but not primary astrocytes (Supplementary Figure S1). Furthermore, we observed that blocking the increase of ROS with the antioxidant NAC and aprocynin resulted in decreased intracellular ROS levels as well as wogonin-induced glioma cell death. These results indicate that ROS accumulation contributes to wogonin-induced apoptosis in human glioma cells. Furthermore, it has also been reported that wogonin-induced cancer cell death through induction of cell apoptosis and ER stress in human sarcoma, but not normal cells [32]. ER stress plays a crucial role in the regulation of apoptosis. Increasing evidence has been reported that caspase activation is involved in ER stress-induced cell apoptosis [13,51,59–61]. Recently, we also demonstrated that phloroglucinol derivatives induces cell apoptosis through ER stress-activated caspases in human colon cancer [42] and glioma cells [62]. However, the interaction between ROS generation and ER stress activation like calpain 1 induction requires further investigations. Our results showed that ROS generation, up-regulation of pro-apoptotic protein and activation of caspases may be involved in wogonin-induced apoptotic cell death in human glioma, with its ability to cause ER stress. In conclusion, wogonin-induced human glioma cell death is mediated by ROS generation, which subsequently induces GPR78 and GRP94 expression, increases caspases activity, such as caspase-9 and caspase-3, resulting in apoptosis. Present study on a molecular basis will provide valuable strategies for wogonin in effective anti-tumor therapy.

## Supplementary Materials
