# Supplementary material for: Wogonin Induces Reactive Oxygen Species Production and Cell Apoptosis in Human Glioma Cancer Cells
Source: Int J Mol Sci. 2012 Aug 8;13(8):9877–92. doi: 10.3390/ijms13089877 (PMC3431834; doi:10.3390/ijms13089877)

Wogonin Induces Reactive Oxygen Species Production and Cell Apoptosis in Human Glioma Cancer Cells

Supplementary Information

Figure S1. Wogonin does not affect ROS generation in human primary astrocytes. Cells were incubated with wogonin (25 μM) for indicated time periods (5, 10, 30, 60 or 120 min). ROS generation was determined using the fluorescence probes H_2DCFH-DA. The production of ROS was examined by flow cytometry. Results of control and wogonin-treated (120 min) group were merged in lower panel.

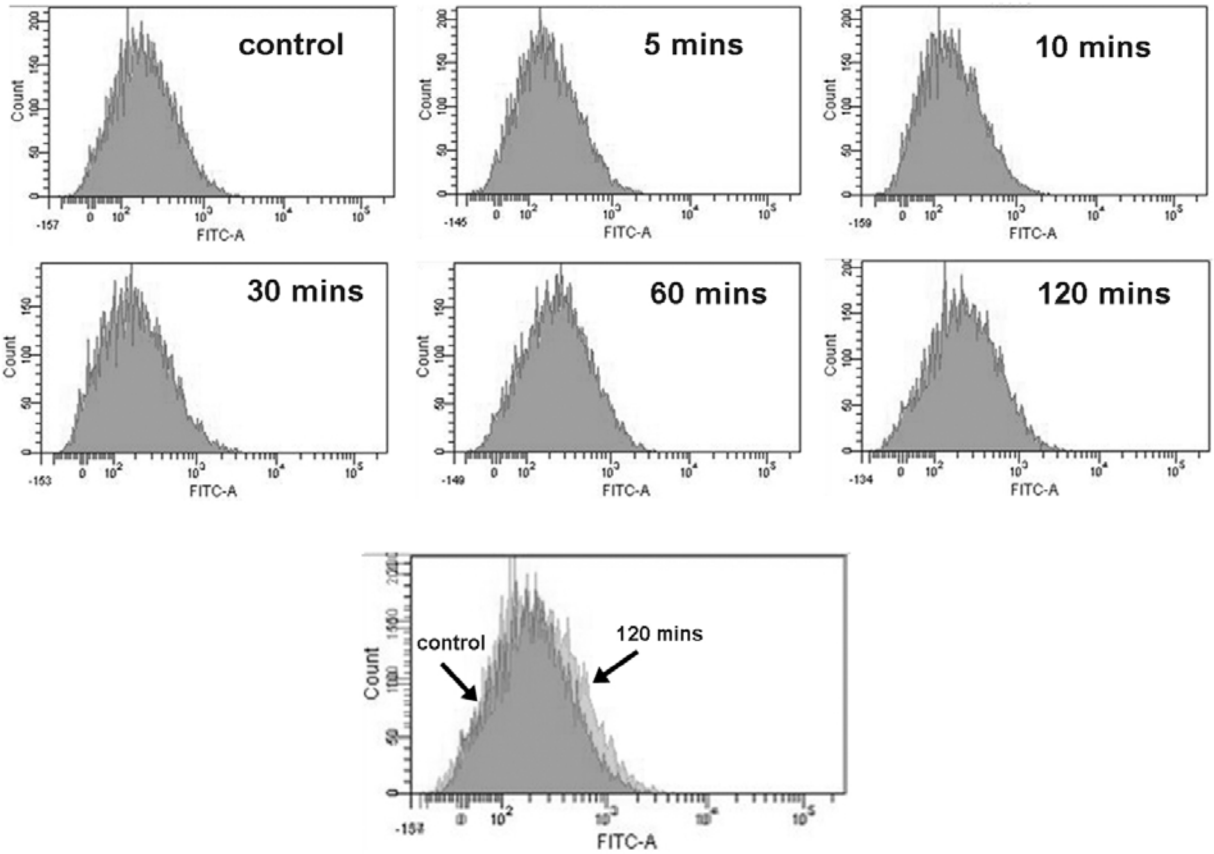

Supplement: Supplementary file 1 [file ijms-13-09877-s001.pdf]
